# *“Balancing work and movement”*: barriers and enablers for being physically active at Indian workplaces – findings from SMART STEP trial

**DOI:** 10.1186/s12966-024-01661-z

**Published:** 2024-09-27

**Authors:** Baskaran Chandrasekaran, Ashokan Arumugam, Arto J Pesola, Chythra R Rao

**Affiliations:** 1 Department of Exercise and Sports Sciences, Manipal College of Health Professions, Manipal Academy of Higher Education, Manipal, Karnataka 576104 India; 2 Department of Physiotherapy, College of Health Sciences, University of Sharjah, Sharjah, United Arab Emirates; 3 Neuromusculoskeletal Rehabilitation Research Group, RIMHS – Research Institute of Medical and Health Sciences, University of Sharjah, P.O. Box: 27272, Sharjah, United Arab Emirates; 4 Sustainable Engineering Asset Management Research Group, RISE - Research Institute of Sciences and Engineering, University of Sharjah, P.O. Box: 27272, Sharjah, United Arab Emirates; 5 Adjunct Faculty, Department of Physiotherapy, Manipal College of Health professions, Manipal Academy of Higher Education, Manipal, Karnataka India; 6 Active Life Lab, South-Eastern Finland University of Applied Sciences, Mikkeli, FI-50100 Finland; 7 Department of Community Medicine, Kasturba Medical College, Manipal Academy of Higher Education, Manipal, Karnataka 576104 India

**Keywords:** Barriers, Office workers, Workplace, Sedentary behaviour, Physical activity, Compliance, Sustainable cities; urban community

## Abstract

**Background:**

Non-communicable diseases are rising rapidly in low- and middle-income countries, leading to increased morbidity and mortality. Reducing sedentary behavior (SB) and increasing physical activity (PA) offer numerous health benefits. Workplaces provide an ideal setting for promoting SB/PA interventions; however, understanding the barriers and enablers is crucial for optimizing these interventions in workplace environments.

**Methods:**

Nested within a cluster randomised controlled trial (the SMART-STEP trial), the present study employed in-depth interviews with 16 office workers who have completed 24 weeks of two distinct (technology assisted and traditional) workplace SB/PA interventions. Using a deductive analysis, semi-structured interviews were administered to explore the barriers and enablers to the SB/PA interventions at individual, interpersonal and organisational level using the socio-ecological model.

**Results:**

Several individual (poor goal setting, perceived health benefits & workload, attitude, intervention engagement), interpersonal (lack of peer support) and organisational (task prioritisation, lack of organisational norm and material or social reward) barriers were identified. Indian women engaged in desk-based office jobs often find themselves burdened with intense home and childcare responsibilities, often without sufficient support from their spouses. A primary concern among Indian office workers is the poor awareness and absence of cultural norms regarding the health risks associated with SB.

**Conclusions:**

Raising awareness among workplace stakeholders—including office workers, peers, and the organization—is crucial before designing and implementing SB/PA interventions in Indian workspaces. Personalized interventions for Indian female office workers engaged in desk-bound work are warranted.

**Supplementary Information:**

The online version contains supplementary material available at 10.1186/s12966-024-01661-z.

## Background

Non-communicable diseases (NCDs), such as cardiometabolic disorders and cancers, account for 74% of global deaths, with cardiovascular diseases being a significant contributor, particularly in low- and middle-income countries (LMICs) including India [1]. Sedentary behavior (SB), defined as any waking activity characterized by low-energy [2], has garnered increasing attention due to its high prevalence in recent times and strong association with chronic disease risk and mortality [3], especially among South Asians [4]. Despite medical advances, reducing SB and improving physical activity (PA) remains crucial for mitigating NCD risk, as emphasized by the WHO’s Global Action Plan [5]. A recent systematic review recommends South Asians should be targeted to reduce sedentary time as their daily SB (≈ 9–10 h) which is comparable to western counterparts [4]. Addressing SB and its associated risks in these LMICs is essential, particularly in settings like workplaces and schools, where a significant portion of waking hours is spent.

Office workers in high-income countries spend 65–80% (> 9 h) of their waking hours in the office, a substantial fraction of which involves prolonged sitting (> 30 min of unbroken bouts) [6, 7]. With the westernization of lifestyles (automation in transportation, household and occupational activities), advances in communication technologies, and the rise of remote sedentary jobs, office workers in LMICs are increasingly experiencing similarly high levels of SB and low levels of PA in their day-today lives [8–10]. However, interventions addressing SB in LMICs remain sparse, partly due to a lack of awareness among organizations and policymakers about the need to develop and implement such interventions [11]. Moreover, Indian workplaces, characterized by cultural diversity, varying workplace policies, and limited economic resources, present unique challenges for SB/PA interventions.

The success of these interventions depends on understanding the specific barriers and facilitators within the Indian context. Emerging evidence from high-income countries suggests that common barriers to SB/PA interventions among office workers include limited capability, negative attitudes towards SB/PA practices, low resources, lack of time, competing task priorities, insufficient environmental and management support, and a generally low PA culture [12–14]. To date, only one cross-sectional study from India has quantitatively synthesized the barriers and facilitators to SB/PA practices among sedentary office workers [15]. The socio-ecological model offers a comprehensive perspective on the complex, interrelated factors at the individual, interpersonal, and organizational levels that influence human health and well-being [16]. Understanding these factors and how they interact is crucial for designing effective interventions. In the context of Indian workplaces, identifying the individual, interpersonal, and organizational determinants that affect adherence to interventions aimed at reducing SB and enhancing PA can guide public health experts in developing and scaling up ecologically valid and culturally adaptable behavior change interventions tailored specifically to these settings [12, 17].

The SMART STEP trial [18] is the first Indian study to investigate the efficacy of a 24-week SB/PA intervention in sedentary Indian workplaces, which are perceived as ideal settings for promoting such interventions. The aim of the present study is to provide insights into the individual, interpersonal, and organizational factors that hinder or support adherence to workplace SB and PA interventions among Indian office workers who participated in the 24-week SMART STEP trial [18].

## Methods

The present semi-structured deductive qualitative study was nested within a randomised controlled trial (SMART STEP trial) [18]. The trial was approved by Institutional Ethics Committee (IEC 2019/746) and prospectively registered in clinical trial registry of India (CTRI/2020/03/024138). The present study followed the ethical guidelines laid by the Declaration of Helsinki and are reported as per the Standards of Reporting Qualitative Research (SRQR) guidelines [19]. Supplementary file S1 shows the SRQR checklist.

### Nature of clusters in SMART-STEP trial

This cluster randomized controlled trial investigated the effects of two behavioural interventions (technology-assisted and traditional workplace interventions) compared to usual work practices among participants in 15 administrative offices within various medical and technological institutions of a multifaceted university in coastal Karnataka. The clusters were stratified based on size (< 10, 10–20, and > 20 employees) with a women-to-men ratio of 2:1. These clusters included offices related to billing, media and marketing, finance and purchasing, record maintenance, and administration within the university’s academic institutions. To be eligible, the participants of the clusters should be a full-time office worker, sedentary & insufficiently active and free from chronic diseases that might limit from adequate PA participation.

The clusters were randomised to either one of three the behavioural arms : (1) SMART intervention: the participants of this group received technology assisted cues to reduce SB during their working hours and improve PA throughout the day; (2) TRADE intervention: the participants of this group received a customised education manual which had information on the risks of SB, benefits and ways to reduce SBs and improve PA both during work and non-work hours compared to (3) CONT group participants who continued their usual work for next six months. In SMART group, the participants received six hourly exercise prompts randomised through customised smartphone application during their working hours. Each prompt lasted for two minutes: one minute of stretching and one minute of calisthenics exercise [18]. If the participant opened the pop-up notification, the session was logged automatically as completed. In addition to prompts during working hours, the participants also received pedometers and were instructed to increase the baseline line step count gradually to 10000 steps and maintain the step count for 24 weeks. The participants of TRADE group received a customised manual which had five modules: dangers of sitting, benefits of PA breaks, strategies to improve PA during working hours and leisure time and dairy logs for reducing sitting and increasing PA at least 150 minutes per week. The TRADE participants were instructed to adhere to the information on the strategies to reduce SB and PA. The ‘high compliant (HC)’ was defined as adherent to at least 70% of the intervention dose (580/828 sedentary breaks assigned during work hours and 101/144 days to 30 min/day of moderate-vigorous PA logged in the smartphone & diary), while ‘low compliant (LC)’ was defined as not adherent to above dose of SB or PA.

### Participants recruited for qualitative study

Semi-structured interviews were conducted among 18 participants who completed the 24 weeks of the SMART-STEP interventions. The participants who were willing to participate in 15–20 min of the interview and express their views on the potential barriers and enablers to the interventions (SMART and TRADE) were selected through purposive sampling. Nine participants from the SMART group (five HC and four LC) and nine participants from the TRADE group (four HC and five LC) of 13 different workplaces situated at different geographical areas of the single university were contacted. While two participants from the TRADE group were not willing (one not willing for her voice to be audiotaped and other denied participation for the fear of shared information with organisation despite the interview being anonymised), the in-depth interviews were conducted for 16 participants and audio recorded. Since no new insights regarding additional barriers to workplace SB/PA practices emerged during the last five interviews, indicating that data saturation had been reached, the sample size was deemed appropriate.

### Interview guide development

**Fig. 1:**
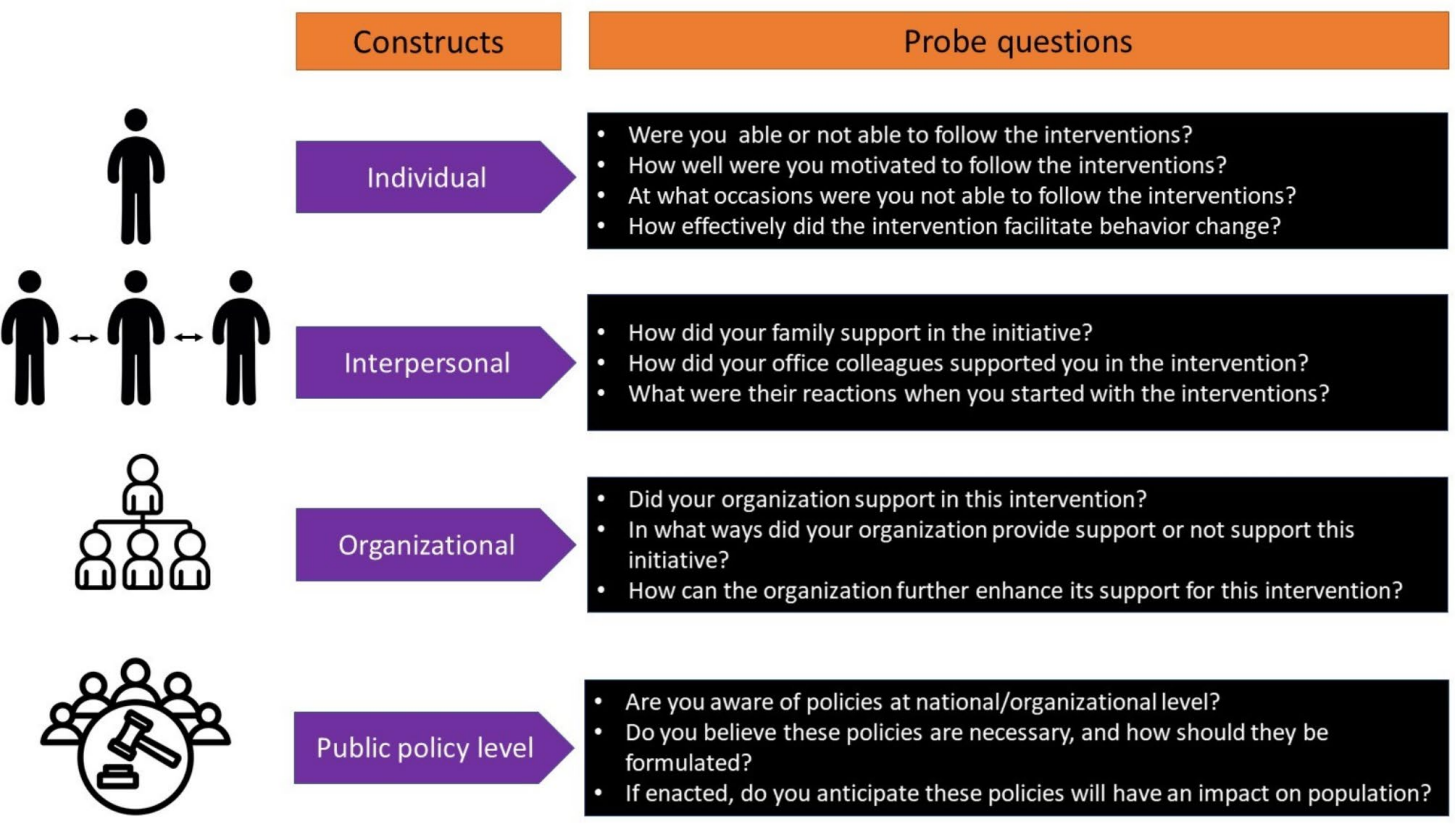
Development of the interview guide based on the socioecological model (individual, interpersonal, organisational and public policy level). The examples of generic questions asked under each domain of the model are shown in the block boxes

A semi-structured interview guide was developed to identify potential barriers and enablers to the two workplace interventions (SMART and TRADE groups) using socioecological model [16]. The interview questions development was guided by the socio-ecological model illustrating the determinants of SB/PA at the individual (biological factors – capability; opportunity, psychological- motivation), interpersonal (social support from family, friends) and organisational (norm, policies, built and environment) [16]. The interview guide is summarized in supplementary file  S2. The questions were developed thematically to elicit responses about the individual, interpersonal, organisational and public policy level barriers and facilitators to workplace interventions (SMART and TRADE) administered in the SMART-STEP trial. The constructs and the questions were chosen a priori based on the previous studies that explored the potential barriers using the socioecological model [20]. Figure 1 depicts the constructs added, and model questions asked in the interview.

Further we added micro-constructs under each main construct of socioecological model: (1) individual (attitude, motivation, capability, opportunity, intervention acceptability); (2) interpersonal (family, colleagues); (3) organisation (managerial support, work/ break policy, workload) and (4) public policy (social norm and policy on labour). Additional constructs identified during the interview and discussion of the excerpts were added during repeated iterations. To explore each construct and micro-constructs, questions were specifically phrased and developed as shown in supplementary file S2 table.

### Interview administration and recording

The interviews were conducted from August to November 2023. The participants were interviewed during their work hours for an in-depth interview in a comfortable private space in the vicinity of the office spaces. The interview was audio recorded using a smartphone (Redmi 9, MIUI version 12.0.18) and saved in Advanced Audio Coding (AAC) format. The AAC files were then imported into the video editing software Clipchamp (Microsoft, US) for auto-transcription with artificial intelligence. The transcription was done in the Indian English language so that erroneous words were avoided. The transcribed audio was then downloaded in rich text format and uploaded in online qualitative research software Taguetee (version 1.4.1-40-gfea8597) [21] for further coding and thematic synthesis.

### Deductive analysis (transcription and coding)

The data was analysed using codebook thematic analysis, a method employed to identify, analyse, and report data patterns, enabling deductive analyses as described in recent qualitative synthesis by Landais (2022) [22] and Hadgraft (2023) [23]. The codebook thematic analysis was done using Taguette web-based tool [21]. The present qualitative synthesis followed the general process of five steps outlined by Castleberry et al., 2018 [24]: (1) *compiling*: the data was transcribed using Clipchamp (Microsoft, US). The transcribed data were compiled and organised by two authors (BC, CRR) for further coding; (2) *disassembling*: the textual data were then separated and identified for common constructs connecting each other by the lead author (BC) using inductive coding approach in the Taguette software online tool [21]. These codes categorised into constructs based on the socioecological model (individual, interpersonal, organisational and policy level) and micro-constructs (e.g., individual – capability, motivation and opportunity, intervention acceptability) by primary author (BC) after discussion with the author (CRR) until mutual agreement about constructs and micro-constructs was reached; (3) *reassembling*: In this step, structured codebook was created in the Taguette web-based tool (version 1.4.1-40-gfea8597) using the above constructs and micro-constructs and constructed into meaningful clusters. For example, the perceived control, attitude, goal setting of the individual were further classified to capability, motivation and opportunity (COM-B) adapted in the behaviour change wheel [25]. COM-B can aid in understanding of the behavior change mechanisms outlined by Socio-ecological model [16]; (4) *interpreting*: the lead author (BC) read and re-read the quotations belonging to each code and established the relation between the constructs and micro-constructs; (5) *concluding*: the participants’ perspectives on barriers to workplace SB and PA intervention were identified, consolidated and summarised. The findings on the barriers and enablers to two workplaces SB and PA interventions (SMART and TRADE) were narratively elaborated. Accuracy of the quotes and the relation with constructs and micro-constructs in the deductive method were re-examined comparing with the excerpts of interview transcripts once compiled.

### Adaption of micro-constructs

**Fig. 2:**
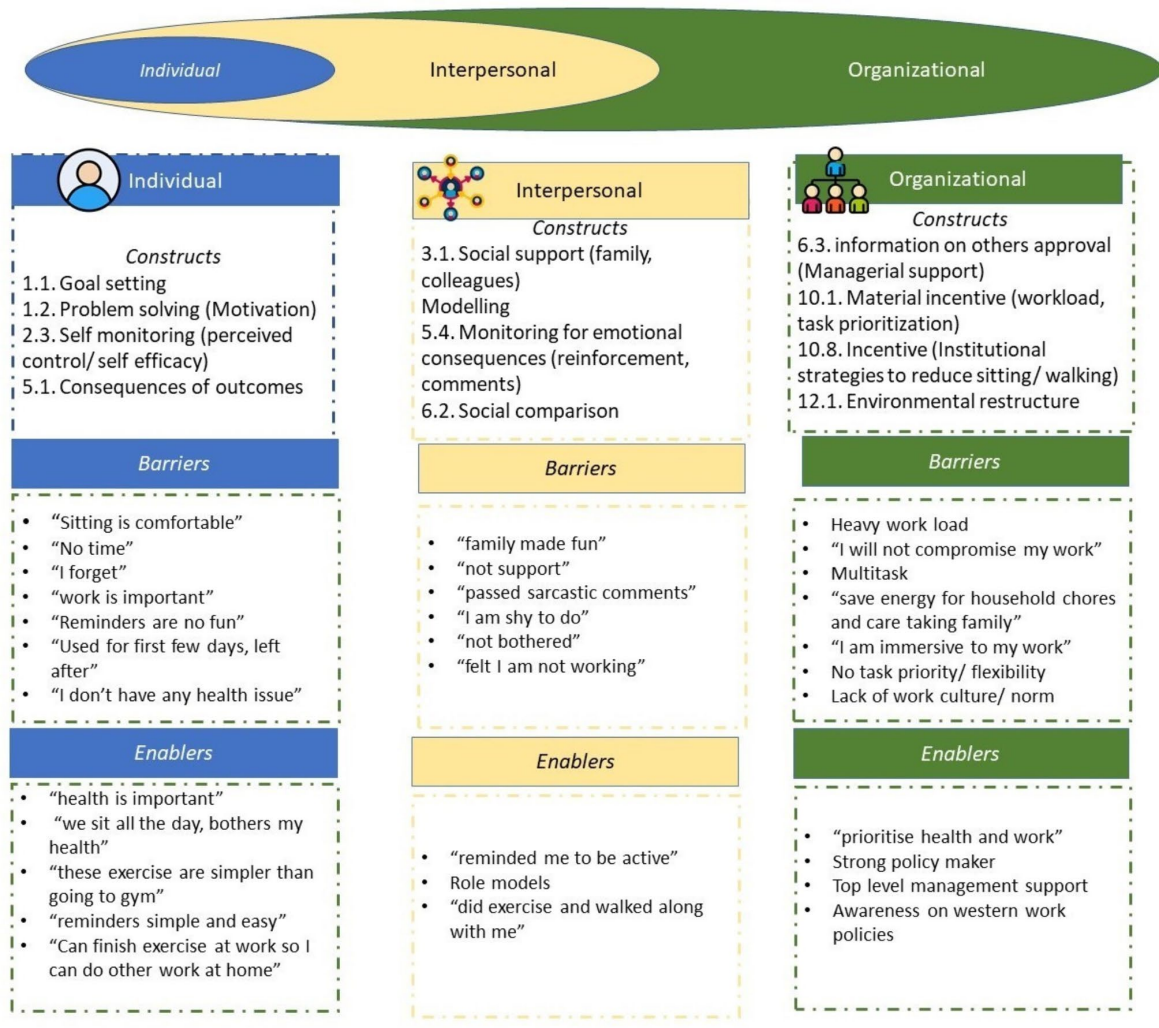
Socioecological framework-based constructs and the perceived barriers and enablers identified in the interview

Initially, the constructs of the socioecological model [16] was used to develop the probing questions to identify the barriers and enablers. However, after coding analysis in the Taguette software open-source tool (version 1.4.1-40-gfea8597) [21], further micro-constructs at individual, interpersonal and organisational levels were identified. Capability, motivation (intrinsic), and opportunity from the theory of behaviour change wheel [16] were adapted at the individual level, while interpersonal and organisational level strategies were grouped to extrinsic motivation. Interpersonal influence was mainly from family (spouse and kids) and colleagues. Micro-constructs of organisational level influence were workload, task priority, managerial support, policies of scheduled breaks and active work, norms and culture, and environmental structure. Figure 2 shows the constructs and micro-constructs identified with the thematic analysis.

### Data analysis

Demographic variables of the participants were presented as median and interquartile range. The interview data were analysed using a deductive thematic analysis in the Taguette web-based tool [21]. The constructs (constructs) were further coded as micro-constructs (micro-constructs) as directed by the socioecological model based which provide a holistic overview for increasing opportunity to improve PA and reducing SB at workplace [16]. Finally, a decision tree was illustrated to decide compliance based on barriers and enablers to the determinants of workplace intervention which may be helpful for public health experts and behavioural scientist to design and map appropriate interventions.

## Results

Of 18 office workers who completed the 24-week intervention trial and invited, 16 office workers (nine SMART group and seven TRADE group participants) participated in the interviews.

### Characteristics of the participants

Of 16 participants, majority were women (*n* = 10, 62.5%) and were senior executives. The participants belong to 9 different institutions of a multifaceted university situated in different geographical areas. Most of the interviewees were from administrative backgrounds and had a shared office space as their place of work. The median age of the participants was 37.5 years and had experience of 9 + years in the current job. The baseline characteristics of participants are illustrated in the Table 1.

**Table 1:** Demographic characteristics of the participants included for the qualitative interview

Characteristics	Value†		
	Overall	SMART	TRADE
*Categorical variables expressed as n (%)*			
Number of participants	16	09 (56)	07 (44)
Gender (women)	09 (63)	06 (60)	04 (40)
Designation (junior executive)	06 (38)	04 (67)	02 (33)
Type of work (administration)	09 (56)	04 (67)	05 (33)
Office cabin (open space)	10 (63)	05 (50)	05 (50)
‘Compliant’ to intervention	9 (56)	05 (56)	04 (44)
*Continuous variables expressed as median (25*,* 75th percentiles)*			
Age (years)	37.5 (34, 42)	36 (34, 42)	38 (35.5, 41.5)
Experience (years)	9.5 (5.8, 14)	8 (6, 14)	11 (8, 13)
Interview time (min)	11.6 (9.2, 14.2)	14.0 (11.0, 14.8)	9.5 (8.6, 12.0)

†median, interquartile range (continuous), number and frequency (nominal)

### Barriers and enablers to workplace interventions

The barriers to workplace intervention (the SMART-STEP trial) among Indian office workers are presented in thematic order: (1) individual, (2) inter-personal, and (3) organisational level. While perceived health threats, family support, and intervention acceptability were viewed as facilitators (a), workload, lack of culture and time, shared workspace, organisational responsibility, and derogatory comments were viewed as major barriers to workplace interventions. Supplementary file S3 & S4 elaborates the barriers and enablers to the SMART-STEP interventions using the constructs and the excerpts from the participants. The quotes are provided for each construct and micro-construct with participant ID, gender, age, low compliant (LC) or high compliant (HC) in parentheses.

#### Individual

Individual factors were found to be the predominant factors influencing adherence to the practice of workplace SB reduction strategies, compared to the interpersonal and the organisational factors. The micro-constructs of the individual factors identified were capability, intrinsic motivation, and opportunity (Supplementary file S3).

#### Opportunity

Majority (n = 10; 62.5%) of the participants perceived less opportunity to practise SB/PA practises at the workplace. These lesser opportunities may be due to perceived higher workload, lack of organisational space or culture to stand or walk during meetings, poor availably of resources were quoted as the prime reasons for the perceived incapability towards the workplace interventions (Supplementary file S3).

Before joining the present institution, I have worked in the corporate sector…. We do get a chance to go and use the gyms or a swimming pool in the working hours….” (P2, F, 36 years, SMART-HC).

*(Q34) “…. during meetings*,* it’s not a culture to even stand and I use to ignore the prompts…. I cannot stand and take notes isn’t it?. Regular desks allow me only for sitting. It (organisational policies) is rigid here….” (P16*,* F*,* 44 years*,* SMART-LC)*.

Specifically, the participants of SMART group perceived that the prompts are rigid without sensing the movement and not engaging such as inclusion of the social networking.

*(Q16) “there is no buddy or group in the app…. If people come together and share their success with colleagues…. Group dynamism…. Something like that… just a prompt…individual entity… It may not work…*”* (P16*,* F*,* 44*,* SMART-LC)*

#### Motivation (intrinsic)

The strong enabler that distinguished between the compliant and non-compliant office workers was identified as intrinsic motivation. The majority of the participants were motivated (*n* = 10, 62.5%), despite the perceived barriers.

*“I strongly believe physical exercise is a must…. Usually what happens is when we get involved with the work*,* we don’t realise how long we have been sitting*,* ……with just a pop up saying you have to move*,* you have to stretch*,* it actually helps. At least a personal reminder for me to move is there it helps.” (P2*,* F*,* 36 years*,* SMART-HC)*.

Few felt that with the tedious workload, urgent tasks, and deadlines, these reminders were helping them to move and prevent musculoskeletal problems caused by their job (Supplementary file S3: Q43-46).

*“You hardly get enough time during work or after work and forget to move physically……. ‘As an office staff*,* I sit almost entire day. We forget to take a break or stretch at least. This intervention is simple reminds me to get off the chair” (P9*,* F*,* 33 years*,* TRADE-HC)*.

*“My health was not up to my expectation even with morning walk. I expected some change during the sitting time in office hours may improve my health benefits” (P1*,* M*,* 47 years*,* SMART-HC)”*.

Though the majority found intrinsic motivation, a few found barriers too. The participants felt that these behaviours would become habits and be sustained only when health risks were perceived (Supplementary file S3: Q4 - Q11).

*“Only when people perceive their health is at risk*,* then only these (interventions) will work. Otherwise its waste of resource*,* money and other resources.” (P14*,* M*,* 36 years*,* TRADE-LC).*

*“First few weeks I was trying…… it didn’t become a habit …… I am comfortable with sitting in my office and working…” (P14*,* M*,* 36 years*,* TRADE-LC)*

#### Intervention acceptability

The majority of the participants felt that the intervention needed more salient features in a contextual setting rather than just simple reminders, which are boring (Supplementary file S3: Q12 - Q16). Participants felt that instead of just rigid notifications, sensing the movement or sitting behaviour first and then appropriately sending exercise prompts (personal approach to end-user goals) would be more beneficial (Supplementary file S3: Q15). Further, the above sensing would be appropriate in the case of attending meetings and unscheduled breaks, which are common in Indian office workspaces.

*“When I am at the meetings and the exercise reminder alarms*,* people think I am setting alarm for moving out…. It looks awkward sometimes.” (P7*,* M*,* 36*,* SMART-LC)*

*“prompt is rigid… if I missed due to meetings and after resumed work*,* it does not sense……. When I want the prompt/clue was not there…. It should sense my sitting time…. Rather only rigid timing prompts…..” (P16*,* F*,* 44*,* SMART-LC)*

Few felt that social networking might help in sharing progress, and compliance might be enhanced with peer support (Supplementary file S1: Q16). Further they expressed that social networking may reduce the derogatory comments during exercise compliance in workplaces.

*“There is no buddy or group in the app…. If people come together and share their success with colleagues…. Group dynamism…. Something like that… Now it’s just a prompt…individual entity… It may not work in long term…*,* (P16*,* F*,* 44*,* SMART-LC)*

Some participants highlighted the benefits of smartphone-based reminders (Supplementary file S3: Q48 - Q53).

*“Now people carry everywhere……It’s (smartphone-based reminders) fun……one stretch based on popup…. Feels good and comfortable……Not monotonous…………smartphone reminders…. different exercise not repeating on the same day…. I liked the idea and did (a) couple of times” (P1*,* M*,* 46 years*,* SMART-HC)*.

### Interpersonal level

#### Family support

All the non-compliant participants expressed concerns about less support from the family. Sometimes the participants have to face the derogatory comments from the spouse and the kids (Supplementary file S3: Q18).

*“………………… they made fun as I started walking in morning and shared the study information to them… They told today ‘you are doing great… but we don’t know how long this is going to last’….” (P7*,* M*,* 36 years*,* SMART-LC).*

However, a few participants felt that they could comply with the workplace interventions due to their family support (Supplementary file S1: Q54-56). The family members were a part of modelling the behaviour and mental support.

*“…………My family was very supportive…….my family members all are kind of sports person… fitter than me” (P13*,* M*,* 42 years*,* SMART-HC)*.

#### Peer support

All the participants expressed the concern of poor peer support in Indian office spaces (Supplementary file S3: Q19 – Q21). The participants have to face the derogatory comments, unpleasant stare from the colleagues who were not the part of the study during initial part of the study. However, the colleagues were least bothered in due course of time.

*“My colleague stared at me once while doing exercise……… She smiled and asked why you are doing all these (exercise) at 11:30 AM… She thought I turned crazy (laughs)…… After that she never bothered”* (*P12*,* F*,* 38 years*,* SMART-HC)*.

*“every individual has their own perception and not everybody will be OK to use*,* you know to get into all these activities……… because of poor awareness of all these things (exercise in office). When I stood to stretch with reminders*,* they use to pass comments*,* however did not bother after that……” (P2*,* F*,* 36 years*,* SMART-HC)*.

Non-compliant participants were sharing common workspaces where they felt shy doing the exercise in front of other colleagues who were not part of the study (Supplementary file S3: Q21).

*“I am shy to do in front of others. It’s an open area you know…. When my cubicle is free…. Just me… I have done the exercises………” (P11*,* F*,* 34*,* TRADE-LC)*.

### Organisational level

Organisational constructs were found to be predominant factors hindering the workplace SB and PA practices among participant of our study (Supplementary file S3: Q22 – Q39). Workload, task priority, organisational norm, policy on active work/ scheduled breaks, managerial support and environmental restructure were identified to be the micro-constructs of the organisational barriers and enablers.

#### Workload

Significant portion of the participants (*n* = 13, 81.3%) were citing the workload as the predominant barrier in practicing scheduled breaks and PA at workplaces (Supplementary file S3: Q22 – Q25).

*“With my typing*,* listening to meeting tasks*,* I was already mentally exhausted. I reserve my physical energy for evening to prepare meals at night and looking after my younger daughter…. I need energy that I did not want to physically exhaust myself with exercises.” (P8*,* F*,* 42 years*,* SMART-LC)*.

Few perceived that these practices would be effective in information technology sectors, where face-to-face interactions with customers and unprecedented tasks were not commonly encountered (Supplementary file S3: Q24). As the study participants were the part of administrative blocks of multifaceted institutions of university, the perceived workload and tasks would be varied. The variation in the work in different office clusters of university makes it difficult to perceive similar workload across the participants.

*“This intervention may work for different institutions which vary in break or work policy. It’s a rigid system in health care accounts sections where task and consumers are priority… It may work for software professionals” (P16*,* F*,* 44 years*,* TRADE-LC)*.

#### Task priority

Most of the participants expressed the priority of the work and the anticipated material reward (monthly salary) for the completion of work rather than reducing SB or improving PA. The majority of participants believed that completing their current work and fulfilling their assigned duties for the organization constituted their identity and contributed to sustainability (Supplementary file S3: Q26, 27). The perceived threat of lethargy or neglecting work was also raised as a concern by a few participants.

*“I immerse so much when I start work…. I ignore even calls…. These mobile notifications……I ignore most…… I want to complete my work assigned to me…. other things come next…” (P13*,* F*,* 37*,* SMART-LC)*.

However, a small number of participants who prioritized their health over their tasks or workload exhibited high compliance with the workplace intervention trial (the SMART-STEP) (Supplementary file S3: Q57).

*“As Indians*,* we do not prioritise things (work and health balance)*,* we take it for granted…. Only when health is at risk*,* they look for such interventions” (P2*,* F*,* 36*,* SMART-HC)*.

#### Managerial demands/ support

Majority of the participants expressed that the managers or workplace champions were least bothered about the performance or adherence towards workplace PA or SB interventions (Supplementary file S3: Q28). The fear of perceived laziness or neglecting work during the working hours by the managers was found to be a significant contributor to the non-adherence in majority of the participants.

*“…. they (managers) are least bothered. We get a chance to do all these (exercise) things only when we get a space for it. If you are not accepted the way you should be then we will be forced to what boss is going to tell us …. least importance is given to the physical fitness or the mental fitness.” (P12*,* F*,* 32 years*,* SMART-HC).*

Few perceived the managers should be aware about the benefits of such SB/PA interventions and authoritative enough to implement in sedentary Indian workspaces (Supplementary file S3: Q58).

#### Active work policies

Participants voiced concerns about the lack of workplace policies promoting physical activity in Indian offices, believing such policies could enhance emotional bonds and productivity (Supplementary file S3: Q29 – Q36). They also stressed the importance of raising awareness among university management and faculty, advocating for initiatives promoting active work, scheduled breaks, flexibility, and recreational facilities to promote exercise and sport.

*“As you know about Indians*,* they take it (health) for granted at young age. We need a push from the institution side……when you come up with certain policies*,* rules and regulations (regarding these kind of exercise at workplace) ……. somewhere you are touching them personally also. Yes*,* institution is concerned about my health…… If I am keeping well*,* I can definitely give back 10 more times.” (P2*,* F*,* 36 years*,* SMART-HC)*.

*“If you want such policy (promoting health in organisations)*,* strong individuals should be there in that position*,* who can implement it*,* and actually see the working process gets on. Unfortunately*,* no one is there to promote such in national level……” (P11*,* F*,* 34 years*,* TRADE-LC).*

*“……. top level organisation should be aware of how more PA will make the employee effective and productive at work. Breaking in-between sitting may actually keep us healthy and more productive” (P13*,* F*,* 37 years*,* SMART-LC).*

**Fig. 3:**
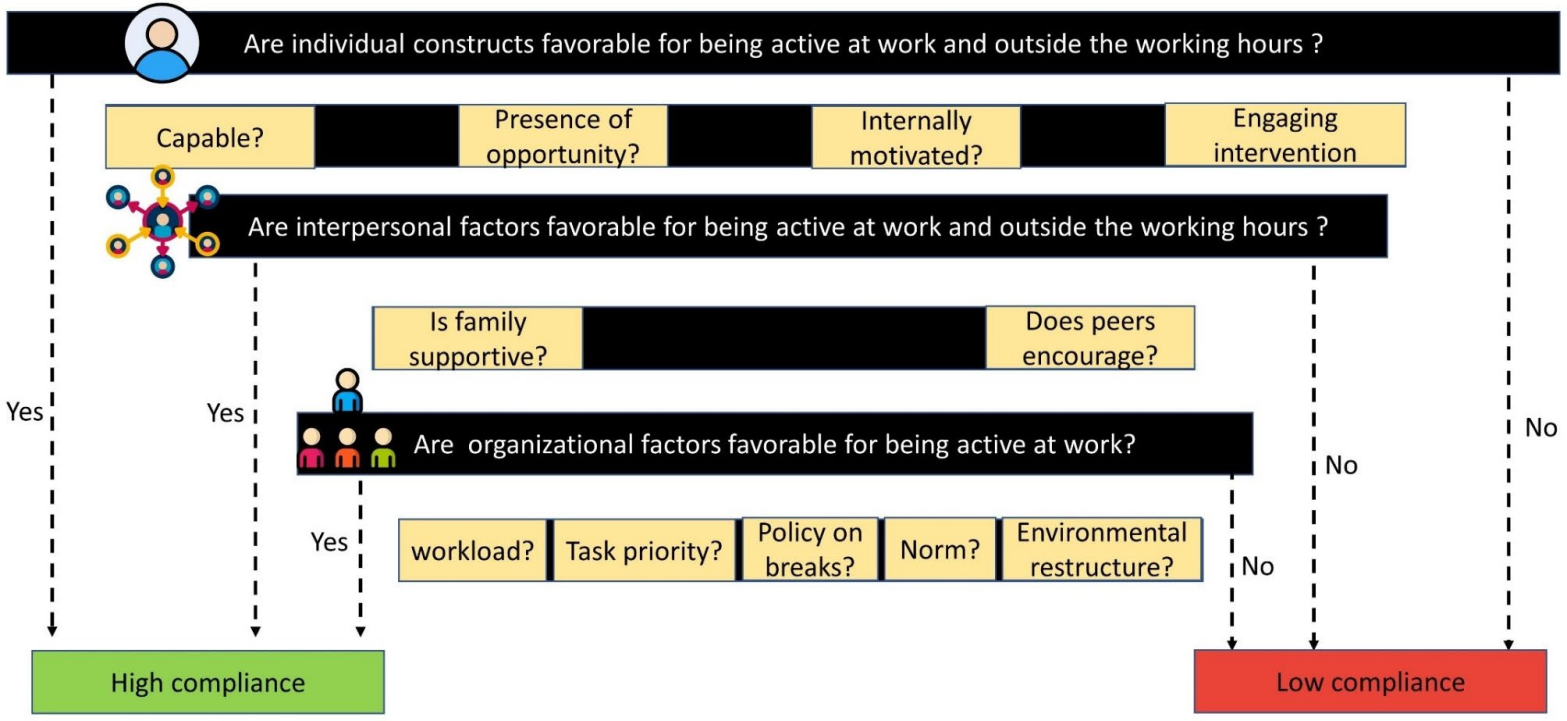
Schematic representation of the algorithm designed to predict adherence among Indian office workers to the intervention, utilizing the constructs and micro-constructs of the socioecological model

Figure 3 depicts the constructs and micro-constructs at the individual, interpersonal and organisational level barriers and enablers to the workplace SB/PA interventions. Supplementary file S4 is the extension of the Fig. 2 demonstrating mind-mapping of constructs and micro-constructs of the barriers and enablers to workplace SB/PA interventions.

### Differences in the barriers/enablers between SMART and TRADE interventions

TRADE group participants perceived more barriers (lack of privacy & policies for scheduled breaks, bored on education-based PA/SB schedules, lack of exercise facilities in the vicinity of working bay) than SMART group participants. Majority of TRADE group participants perceived organisation-based barriers are more significant than individual level barriers (Supplementary file S3).

*(Q31) “In case you have a job where you have to sit for …. say 9 hours*,* 10 hours*,* the management should be flexible in allowing for breaks or exercise or gym provisions” (P11*,* F*,* 34 years*,* TRADE-LC).*

*Q37 - “providing exercise facilities at workplace may encourage more rather than simple break schedule… I am bored”* (*P5*,* M*,* 39 years*,* TRADE-LC)*.

*Q60 “these things possible only when the organisations make it compulsory for these kinds of…. practicing exercise or walk breaks…. during office hours……” (P4*,* F*,* 43*,* TRADE-LC).*

On the other hand, SMART group participants perceived individual barriers (not using mobile phones during work, not engaging and rigid interventions) and interpersonal (perceived neglect of work, sarcastic comments on alarms) more than the TRADE group participants.

*(Q12) “See my work is completely computer based and liaison with the administrative representatives*,* I do not have time to check the mobile only for exercise break videos. I do not see mobile phone sometimes hours together due to workload” (P6*,* F*,* 39 years*,* SMART-LC)*.

*(Q13) “I didn’t follow because it’s just an alarm…. that tells me what I must do.” (P8*,* F*,* 42 years*,* SMART-LC)*.

*(Q14) “When I am at the meetings and the exercise reminder alarms*,* people think I am setting alarm for moving out…. It looks awkward sometimes.” (P7*,* M*,* 36*,* SMART-LC)*

*(Q15) “prompt is rigid… if I missed due to meetings and after resumed work*,* it does not sense……. When I want the prompt/clue was not there…. It should sense my sitting time…. Rather only rigid timing prompts….”. (P16*,* F*,* 44*,* SMART-LC)*

## Discussion

Our qualitative interviews, nested within the randomized controlled trial (SMART-STEP), aimed to identify the potential barriers and enablers to reducing sedentary behavior (SB) and promoting physical activity (PA) among Indian office workers. Key barriers identified included a lack of awareness about SB/PA practices, less engaging intervention strategies, shared workspaces, lack of organisational norm insufficient peer support, and the absence of incentives for maintaining fitness at work. Notably, the study’s predominance of female participants, who often juggle high familial and domestic responsibilities, further compounded the low uptake of SB/PA practices due to the blurred boundaries between work and life.

While intrinsically motivated, a significant proportion of the present study participants found themselves unable to perform SB/PA interventions at workplace due to perceived work priorities, unexpected meeting obligations, absence of material rewards for doing exercise at workplace, and a lack of specific organizational policies (standing meetings, lunch walk and organised sports). Our study findings align with a recent review that synthesized evidence from 12 quantitative studies, concluding that similar barriers are prevalent in workplaces in high-income countries [26]. Lack of organisational culture/ norm, prioritisation of the tasks over health, absence of material reward for being physically fit and organisational interest on completion of task rather than promoting PA at workplaces are perceived as common barriers to SB/PA practises in Indian workplaces in contrary to their western counterparts [22]. The present study depicts that providing rigid digital solutions which involved simple break reminders or step counts alone may not enhance the behavior change or habit formation unless supplemented with intelligent environment-based sensing systems [27]. Emerging evidence claims that behavioral change can be possible if the digital interventions are developed with features of gamification, social networking and social reward [28]. Further the interventions administered to the study were not individually tailored to the needs of the participants [29]. To be successful, digital and educational interventions should be developed based on participatory approaches and should be co-designed with the stakeholders (office workers, managers, public health experts and developers) [30]. Our interventions were developed by the joint efforts of public health experts and digital solution developers but did not involve the office workers or organizational personnel. Further, the evidence emerging from high income countries found the promising results when the digital interventions were complemented with change in physical environment (i.e., installing active workstations) [31].

Respondents who were compliant have expressed the significant support of their family members, while non-compliant participants have not found the same. Family support on PA promotion has been recognised for decades, however the influence on workplace is less recognised [32, 33]. Emerging studies from low- and middle-income countries (LMICs) highlight the significant role of family in habit formation, particularly in promoting physical activity [34]. However, evidence on workplace behaviors remains scarce. When it comes to peer support, encountering derogatory comments and unpleasant stares from colleagues is not uncommon in Indian workspaces, where tasks or jobs are often prioritized, and awareness about workplace SB/PA practices is relatively low [35]. This is less commonly seen in high income countries where the awareness about ill effects of sitting is largely known [20].

Few of our study participants felt that the break reminders gave them opportunity to move around, talk to others and socialise themselves during office hours for a while. This social interface opportunity with the workplace interventions is observed earlier by Bredahl (2015) [20]. However, these communications have not influenced other peers to participate or compliance in the intervention in contrary to the findings by Bredahl (2015) [20]. Organisational level constructs including cultural micro-constructs (values and beliefs) are found to be the crucial determinants of sustaining workplace PA and SB practices among Indian office workers [36]. Majority of our participants perceived the busy work schedule, intense and unprecedented workload which is further reinforced by the lack of flexibility to break and integrate activities during working hours. This finding is similar to the previous literature which also found workload and lack of flexibility to exercise at the workplace [20].

The barriers commonly encountered during SB/PA implementation at workplaces of LMICs which are culturally and socio-economically diverse from high income countries and among each other, are: lack of time (Congo) [37], fatigue, lack of single-sex gyms, uncomfortable with gym clothing during working hours, not participate in exercise activities in the presence of men (Afghanistan) [38], long working hours (> 10 h) and lack of support, partner, exercise facilities and harassment from colleagues (Bangladesh) [39], fear of injuries, lack of qualified managers, unattractive exercise/ workstation facilities, lack of support from employers, lack of skills in sports and recreational activities (Nigeria) [40], exhausted with day long work, uncomfortable workstations, decreased job satisfaction, presence of chronic disease with limitation on PA (Iran) [41], difficulties in technology access, trips associated with work, discouraging goal reaching efforts (Korea) [42], living in urban area, cultural factors (Nepal) [43], gender difference (Pakistan) [44] (Philippines), office distance (Papua New Guinea) [45], education/ awareness, young age of perceived low risk of diseases (Tunisia) [46], transportation infrastructure to office (Zambia and Zimbabwe) [47]. Our findings align with the existing literature on several barriers, such as reluctance to engage in PA in the presence of others, cultural influences, lack of gym facilities, exhaustion from a long workday, limited education and awareness, a perceived low risk of disease among younger employees, long working hours, and insufficient employer support. However, unlike office workers in other low- and middle-income countries, Indian office workers do not express significant concerns about travel or fear of injuries.

Additionally, Indian women office workers expressed concerns about working throughout the day, including responsibilities such as cooking, caregiving, educating their siblings, and household-financial management outside of working hours, leaving them with no leisure time. The multiple roles adapted by Indian working women along with childcare and household labour and somatic anxiety due to work-family interface have been documented by Rout et al., who surveyed domestic responsibility, job satisfaction, mental health and job stressors among 50 career women from India and England [48]. Our study aligns with the findings of the same study which reported that women from India tend to suppress anxiety and exhibit more somatic symptoms compared to their Western counterparts [48]. Additionally, only a few women expressed shared responsibility for household and childcare duties with their spouses [48].

Based on the presence of above constructs and micro-constructs evolved, a handy algorithm was devised to aid the organisational stakeholders to determine the compliance of its office workers to SB/PA practices (Fig. 3).

Our study is the first of its kind to explore the barriers and enablers to SB/PA practices in workplace implemented among desk-based office workers in India, where rigid work systems and limited awareness of workplace health and hygiene prevail. Moreover, our study paves the way for further research in India to explore the experiences of office workers and organizational barriers that may contribute to the reinforcement of workplace SB/PA interventions in Indian contexts. Our results should also be interpreted in light of following limitations: (1) the perceived barriers and enablers to the workplace interventions belong to a small cohort of office workers belonging to single university [9, 18]. Though the views explain the cultural variability of the Indian office workers perceptions on PA, the readers are cautioned against the generalised interpretation. Further, India is a land of cultural diversity and hence our study results vary between the states too; (2) the PA and SB interventions implemented in the randomised controlled trial in which the present qualitative interview is nested was initially designed on individual level behaviour change theories (social cognitive model) without including the organisational constructs to improve SB/PA in workplaces. Previous SB reduction interventions have reported limited effectiveness for individual-level behavioural strategies during working hours, as compared with better effectiveness for individual and family-level strategies outside of working hours [33].

## Conclusions

Our qualitative study findings indicated that organisational factors such as task prioritisation, workload, lack of social norm/culture, lack of autonomy and task flexibility in workspaces for integration of recreational PA to workspaces were perceived as barriers for implementing and sustaining the workplace PA and SB interventions in Indian workspaces. Further interpersonal factors like family and peer support also seemed to contribute to adherence to workplace SB/PA interventions in the long term. Raising awareness among key workplace stakeholders (office workers, managers, and top management professionals) about the benefits of incorporating SB breaks and PA practices into their work routine is crucial. This awareness can significantly enhance the adoption and sustained adherence to such PA practices, fostering a culture of long-term health benefits among Indian office workers.

## Electronic supplementary material

Below is the link to the electronic supplementary material.

Supplementary Material 1

## Data Availability

All the available data was presented in the study. The main Taguettee coding data for the deductive analysis will be available on reasonable request to the corresponding author. After the corresponding author gets the approval of other authors, the data will be shared to the requesting party with reservations.

## Code Availability

Not available.

## Abbreviations

AAC: Advanced Audio Coding
CONT: Control group
HC: High Compliance
LC: Low Compliance
LMIC: Low-middle income countries
NCD: Non-Communicable Diseases
PA: Physical Activity
SB: Sedentary Behavior
SMART: Technology based intervention group
SRQR: Standards of Reporting Qualitative Research
TRADE: Traditional education-based intervention group
WHO: World Health Organisation
