# Supplementary material for: “Balancing work and movement”: barriers and enablers for being physically active at Indian workplaces – findings from SMART STEP trial
Source: Int J Behav Nutr Phys Act. 2024 Sep 27;21:110. doi: 10.1186/s12966-024-01661-z (PMC11438046; doi:10.1186/s12966-024-01661-z)

***“Balancing work and movement”: Barriers and enablers for being physically active at Indian workplaces – Findings from SMART STEP trial***

**Supplementary files**

Supplementary file S1: Standards for Reporting Qualitative Research (SRQR)

Supplementary file S2. Semi-structured qualitative interview guide

**Supplementary file S3.** Themes, subthemes and excerpts from the participants for barriers & enablers for physical activity interventions at workplace

**Supplementary file S4.** Mind map explaining the barriers and facilitators to workplace PA interventions among participants who have completed the 24 weeks of SMART-STEP trial.

Supplementary file S1: Standards for Reporting Qualitative Research (SRQR)

| No | Topic | Item | Page |
| --- | --- | --- | --- |
| **Title and abstract** | | |  |
| S1 | Title | Concise description of the nature and topic of the study Identifying the study as qualitative or indicating the approach (e.g., ethnography, grounded theory) or data collection methods (e.g., interview, focus group) is recommended | 1 |
| S2 | Abstract | Summary of key elements of the study using the abstract format of the intended publication; typically includes background, purpose, methods, results, and conclusions | 1 |
| **Introduction** | | | 2 |
| S3 | Problem formulation | Description and significance of the problem/phenomenon studied; review of relevant theory and empirical work; problem statement | 2 |
| S4 | Purpose or research question | Purpose of the study and specific objectives or questions | 2 |
| **Methods** | | | 3 - 7 |
| S5 | Qualitative approach and research paradigm | Qualitative approach (e.g., ethnography, grounded theory, case study, phenomenology, narrative research) and guiding theory if appropriate; identifying the research paradigm (e.g., postpositivist, constructivist/interpretivist) is also recommended; rationaleb | 5-7 |
| S6 | Researcher characteristics and reflexivity | Researchers’ characteristics that may influence the research, including personal attributes, qualifications/experience, relationship with participants, assumptions, and/or presuppositions; potential or actual interaction between researchers’ characteristics and the research questions, approach, methods, results, and/or transferability | 6 |
| S7 | Context | Setting/site and salient contextual factors; rationale | 5 |
| S8 | Sampling strategy | How and why research participants, documents, or events were selected; criteria for deciding when no further sampling was necessary (e.g., sampling saturation); rationale | 21 |
| S9 | Ethical issues pertaining to human subjects | Documentation of approval by an appropriate ethics review board and participant consent, or explanation for lack thereof; other confidentiality and data security issues | 21 |
| S10 | Data collection methods | Types of data collected; details of data collection procedures including (as appropriate) start and stop dates of data collection and analysis, iterative process, triangulation of sources/methods, and modification of procedures in response to evolving study findings; rationale | 22 |
| S11 | Data collection instruments and technologies | Description of instruments (e.g., interview guides, questionnaires) and devices (e.g., audio recorders) used for data collection; if/how the instrument(s) changed over the course of the study | 22-24 |
| S12 | Units of study | Number and relevant characteristics of participants, documents, or events  included in the study; level of participation (could be reported in results) | 23 |
| S13 | Data processing | Methods for processing data prior to and during analysis, including transcription, data entry, data management and security, verification of data integrity, data coding, and anonymization/deidentification of excerpts | 23 |
| S14 | Data analysis | Process by which inferences, themes, etc., were identified and developed, including the researchers involved in data analysis; usually references a specific paradigm or approach; rationale | 24 |
| S15 | Techniques to enhance trustworthiness | Techniques to enhance trustworthiness and credibility of data analysis (e.g., member checking, audit trail, triangulation); rationaleb | 24 |
| **Results/findings** | | | 4-15 |
| S16 | Synthesis and interpretation | Main findings (e.g., interpretations, inferences, and themes); might include development of a theory or model, or integration with prior research or theory | 4 |
| S17 | Links to empirical data | Evidence (e.g., quotes, field notes, text excerpts, photographs) to substantiate  analytic findings | 8-15 |

| **Discussion** | | | 16-20 |
| --- | --- | --- | --- |
| S18 | Integration with prior work, implications, transferability, and contribution(s) to the field | Short summary of main findings; explanation of how findings and conclusions connect to, support, elaborate on, or challenge conclusions of earlier scholarship; discussion of scope of application/ generalizability; identification of unique contribution(s) to scholarship in a discipline or field | 16-19 |
| S19 | Limitations | Trustworthiness and limitations of findings |  |
| **Other** | | |  |
| S20 | Conflicts of interest | Potential sources of influence or perceived influence on study conduct and  conclusions; how these were managed | NA |
| S21 | Funding | Sources of funding and other support; role of funders in data collection, interpretation, and reporting | NA |

O'Brien BC, Harris IB, Beckman TJ, Reed DA, Cook DA. Standards for reporting qualitative research: a synthesis of recommendations. Acad Med 2014 Sep;89(9):1245-51.

aThe authors created the SRQR by searching the literature to identify guidelines, reporting standards, and critical appraisal criteria for qualitative research; reviewing the reference lists of retrieved sources; and contacting experts to gain feedback. The SRQR aims to improve the transparency of all aspects of qualitative research by providing clear standards for reporting qualitative research. bThe rationale should briefly discuss the justification for choosing that theory, approach, method, or technique rather than other options available, the assumptions and limitations implicit in those choices, and how those choices influence study conclusions and transferability. As appropriate, the rationale for several items might be discussed together.

**Supplementary file S2.** Semi-structured qualitative interview guide

Participant ID:__________________Date of interview:____________Total time required: 20-30 min

Age: _________ years Gender  Male  female Years of experience: _______ years

Institution of work: _________________________________________________________________

Intervention completed: SMART TRADE Compliance: High (>50%) Low >50%)

**Introduction**

- Introduction by researcher
- Participant signs informed consent
- Researcher explains the structure of the interview
- Recording starts

Thank you for your willingness to participate in an interview about identifying the barriers and facilitators to the 24-week interventions aimed to reduce sedentary behavior and improving physical activity in office workers.

You have now completed the 24-week intervention to reduce your sedentary time. We’d like to ask a few questions about your experience with the interventions. Your responses are extremely valuable in helping us and other researchers develop similar interventions in the future. There are no right or wrong answers. Please be honest about your behavior.

--------------------------------------------------------✂-----------------------------------------------------

The following is questions is for **SMART group participants**

| Components | Micro components | Questions to be asked | Probe |
| --- | --- | --- | --- |
| Individual | Attitude | What are the factors made you to participate in the intervention? | Information about Sedentary behavior and health at workplace? |
|  |  | Have you attempted to add lifestyle change (reduce sedentary behavior or improve physical activity) at workplace | Can you elaborate more?  Do you use treadmill, smart watch? |
|  |  |  | lunch break walks at workplace? |
|  |  |  | Did you find any challenges or obstacles in practicing |
|  |  | Anytime you find challenges in breaking up sitting? | Meetings, important tasks |
|  |  | Any benefits/ hazards with reducing sitting you experienced | Musculoskeletal pain, feel mood, vigor at work |
|  | Perceived control | Were you able to comply to the mobile prompts? | What factors for non-compliance – time, task priority, not demanding? |
|  |  |  | What factors do you like? (engaging, health perspective) |
| Interpersonal relation | Family | Did your family support was there to involve in this project? | Walking support with pedometers, help in step-based intervention? |
|  |  |  | The family members (any) modeled for physical activity participation. |
|  | Peers at workplace | Did you find any support or sarcasms from your peers? | Have your peers involved in the workplace physical activity intervention? |
|  |  |  | How they helped or stopped you to involve in the technology-based intervention |
|  |  |  | Any derogatory comments on your engagement in app or workplace physical activity? |
| Community (organizational) | Task and managers | Do your workplace allow for breaking up sitting | How your workplace aided in your intervention? Any policy in place? |
|  |  | Did your manager/ workplace champion encouraged for your workplace sedentary behavior? | Task priority, modelling, any other encouragements |
| Societal norm |  | Have you heard about workplace policies regarding sedentary behavior and physical activity? | Presence of active workstations, use of stairs, parking policies or office architectural barriers |

Now I’d like to talk about your experience with the smartphone-based prompts, pedometer-based walk intervention and how it influenced your ability to decrease or break up your sitting.

|  | Questions to be asked | Probe |
| --- | --- | --- |
| Smartphone application prompts | Do you feel your prompts engaging? Explain | Liking, not interesting, annoying, not customized? |
|  | Do you feel problem with the phones? | Battery, crash, data/server problems |
|  | In what way we can improve the app that can motivate you? | Games, any sensors integrated |
|  | What will motivate you to increase your physical activity? | Messages, information, absence of other prompts (organizational, availability of treadmill, breaks) |
| Pedometer based step intervention | How difficult was it for you to increase your daily steps by 700 per week or 10000 steps per day?” | Time, comfort, task priority, absence of health problems, |

**The following is questions is for TRADE group participants**

| Components | Micro components | Questions to be asked | Probe |
| --- | --- | --- | --- |
| Individual | Attitude | What are the factors made you to participate in the intervention? | Information about Sedentary behavior and health at workplace? |
|  |  | Have you attempted to add lifestyle change (reduce sedentary behavior or improve physical activity) at workplace | Can you elaborate more?  Do you use treadmill, smart watch? |
|  |  |  | lunch break walks at workplace? |
|  |  |  | Did you find any challenges or obstacles in practicing |
|  |  | Anytime you find challenges in breaking up sitting? | Meetings, important tasks |
|  |  | Any benefits/ hazards with reducing sitting you experienced | Musculoskeletal pain, feel mood, vigor at work |
|  | Perceived control | Were you able to comply to the education manual information? | What factors for non-compliance – time, task priority, not demanding? |
|  |  |  | What factors do you like? (engaging, health perspective) |
| Interpersonal relation | Family | Did your family support was there to involve in this project? | Walking support with 30 minutes of everyday walk |
|  |  |  | The family members (any) modelled for physical activity participation (walking together) |
|  | Peers at workplace | Did you find any support or sarcasms from your peers? | Have your peers involved in the workplace physical activity intervention? |
|  |  |  | How they helped or stopped you to involve in the education based physical activity intervention? |
|  |  |  | Any derogatory comments on your engagement in app or workplace physical activity? |
| Community (organizational) | Task and managers | Do your workplace allow for breaking up sitting? | How your workplace aided in your intervention? Any policy in place? |
|  |  | Did your manager/ workplace champion encouraged for your workplace sedentary behavior? | Task priority, modelling, any other encouragements |
| Societal norm |  | Have you heard about workplace policies regarding sedentary behavior and physical activity? | Presence of active workstations, use of stairs, parking policies or office architectural barriers |

Now I’d like to talk about your experience with the education booklet – strategies to reduce sedentary behavior and increase 30 minutes of physical activity daily.

|  | Questions to be asked | Probe |
| --- | --- | --- |
| Active breaks (exercises mentioned) in the manual | Are you able to do or engage in the exercise during the breaks? How you feel? | Liking, not interesting, not engaging? |
|  | What will motivate you to increase your physical activity? Any other information lacking? | Messages, information, absence of other prompts |
| ACSM Based exercise prescription | How difficult was it for you to engage in 30 minutes of everyday or 150 minutes weekly | Time, comfort, task priority, absence of health problems, |

**End of the interview:** Would you like to add anything we have not yet discussed but might be of relevance for this interview? Do you have any further questions?

--------------------------------------------end of the interview--------------------------------------------

**Supplementary file S3.** Themes, subthemes and excerpts from the participants for barriers & enablers for physical activity interventions at workplace

| Major construct | Subthemes/  constructs | Excerpts | |
| --- | --- | --- | --- |
| **Barriers to intervention** | | | |
| Individual | Opportunity | *(Q1) “If I want to stand during meetings, others may think I am in rush to leave…...Organisations should create opportunities to allow for flexible movements during official hours…. (P3, M, 42 years, SMART - HC)* | |
|  |  | *(Q2) “during 9-5, I am supposed to reply mails, sort problems and provide client feedback…. How can you expect me not to miss few of the prompts….? (P6, F, 39 years, SMART G - LC)* | |
|  |  | *(Q3) “I feel these (exercise or walk inside office) may not work in Indian office spaces. Organised sports or subsidised gyms, which employees can access after work hours or leisure hours may work…” (P7, F, 36 years, SMART - LC)* | |
|  | Intrinsic motivation/ attitude/ awareness | *(Q4) “………Only when people perceive their health is at risk, then only these (interventions) will work. Otherwise its waste of resource, money and other resources. (P14, M, 36 years, TRADE-LC)”* | |
|  |  | *(Q5) “…. I have low back pain due to my long sitting periods, I definitely understand but something hindered me to follow the intervention. I tried in the first few weeks, however left…………….” (P8, F, 42 years, SMART-LC)* | |
|  |  | *(Q6) “I have back pain, obesity and high blood pressure problem…. I have family problem of high sugar levels; however, I am not able to do……... because I am comfortable sitting…. (*P*8, F, 42 years, SMART-LC)”* | |
|  |  | *(Q7) “over a period of time ………. we are unable to completely adhere to the reminders. But if one has ill-health, may be able to follow-up for long term”. (P14, F, 42 years, SMART-LC)* | |
|  |  | *(Q8) “I wanted to see whether it’s (intervention) really helping me because I know I'm sedentary because of the kind of work. I don't have additional time for exercise or anything like that.” (P6, F, 39 years, SMART-LC).” (P14, F, 42 years, SMART-LC)* | |
|  |  | *(Q9) “I know that very clearly, I should not be sedentary … knowing that I am from a family of diabetics, I know all those things, but still something is stopping may be laziness, may be like as I said more career oriented and want to finish my task, my priorities.”* (*P8, F, 42 years, SMART-LC)* | |
|  |  | *(Q10) “I don’t have time to do exercise in morning, mechanical life (laughs)……I thought it (smartphone) gives me simple exercises so can finish during the worktime so that I may not walk or exercise in evening…... I tried you know…. For the first few weeks, but somehow missed the track.”* (*P6, F, 39 years, SMART-LC)* | |
|  |  | *(Q11)* *“First few weeks I was trying…… it didn’t become a habit …... I am comfortable with sitting in my office and working… (P14, M, 36 years, TRADE-LC))* | |
|  | Intervention acceptability | *(Q12) “See my work is completely computer based and liaison with the administrative representatives, I do not have time to check the mobile only for exercise break videos. I do not see mobile phone sometimes hours together due to workload” (P6, F, 39 years, SMART-LC)* | |
|  |  | *(Q13) “I didn’t follow because it’s just an alarm…. that tells me what I have to do.”* (*P8, F, 42 years, SMART-LC)* | |
|  |  | *(Q14) “When I am at the meetings and the exercise reminder alarms, people think I am setting alarm for moving out…. It looks awkward sometimes. (P7, M, 36, SMART-LC)* | |
|  |  | *(Q15) “prompt is rigid… if I missed due to meetings and after resumed work, it does not sense……. When I want the prompt/clue was not there…. It should sense my sitting time…. Rather only rigid timing prompts….”. (P16, F, 44, SMART-LC)* | |
|  |  | *(Q16) “there is no buddy or group in the app…. If people come together and share their success with colleagues…. Group dynamism…. Something like that… just a prompt…individual entity… It may not work…, (P16, F, 44, SMART-LC)* | |
|  |  | *(Q17) “I reach home late evening….7:30 – 8.00 PM…. this pedometer thing…. Rarely I notice…. I used for few days initially…. (P13, F, 37 years, SMART-LC)* | |
| Interpersonal | Family support | Extrinsic motivation | *(Q18) ………………… they made fun as I started walking in morning and shared the study information to them… They told “today you are doing great… but we don’t know how long this is going to last…. (P7, M, 36 years, SMART-LC). …* |
|  | Peer perception |  | *(Q19) “My colleague stared at me once while doing exercise……. She smiled and asked why you are doing all these (exercise) at 11:30 AM… She thought I turned crazy (laughs)…. After that she never bothered”* (*P12, F, 38 years, SMART-HC)* |
|  |  |  | *(Q20) “every individual has their own perception and not everybody will be OK to use, you know to get into all these activities……. because of poor awareness of all these things (exercise in office). When I stood to stretch with reminders, they use to pass comments, however did not bother after that….” (P2, F, 36 years, SMART-HC)* |
|  |  |  | *(Q21) “I am shy to do in front of others. It’s an open area you know…. When my cubicle is free…. Just me… I have done the exercises………” (P11, F, 34, TRADE-LC)* |
| Organisational | Workload |  | *(Q22) “With my typing, listening to meeting tasks, I was already mentally exhausted. I reserve my physical energy for evening to prepare meals at night and looking after my younger daughter…. I need energy that I did not want to physically exhaust myself with exercises.” (*P*8, F, 42 years, SMART-LC)* |
|  |  |  | *(Q23) “from 9 – 5 our work is to type & arrange papers, communicating emails 2-3 hours at a stretch, we don’t find time or forget to do, (P1, M47, SMART-LC)* |
|  |  |  | *(Q24) “This intervention may work for different institutions which vary in break or work policy. It’s a rigid system in health care accounts sections where task and consumers are priority… It may work for software professionals” (P16, F, 44 years, TRADE-LC)* |
|  |  |  | *(Q25) “workplace sitting means it depends your demand or task……time management……. the people are aware of it (reducing sitting in office), but certain sometimes the work task will make them to sit for a longer time…. you can’t just avoid it” (P5, M, 39 years, TRADE-LC)* |
|  | Task priority |  | *(Q26) “You know, I've visited a physiotherapist several times for back pain, and they suggested moving every hour when sitting. The intervention is good in that regard. But my laziness and work priorities... I'm quite comfortable with my chair and sitting." (Participant 8, Female, 42 years, SMART-LC)* |
|  |  |  | *(Q27) I immerse so much when I start work…. I ignore even calls…. These mobile notifications…...I ignore most…… “I want to complete my work assigned to me…. other things come next…” (P13, F, 37, SMART-LC)* |
|  | Managerial demands/support |  | *(Q28) “…. they (managers) are least bothered. We get a chance to do all these (exercise) things only when we get a space for it. If you are not accepted the way you should be then we will be forced to do what boss is going to tell us …. least importance is given to the physical fitness or the mental fitness.” (P12, F, 32 years, SMART-HC)* |
|  | Policies on breaks/ active work |  | *(Q29) “As you know about Indians, they take it (health) for granted at young age. We need a push from the institution side……when you come up with certain policies, rules and regulations (regarding these kind of exercise at workplace) ……. somewhere you are touching them personally also. Yes, institution is concerned about my health…... If I am keeping well, I can definitely give back 10 more times.” (P2, F, 36 years, SMART-HC)* |
|  |  |  | *(Q30) “if provisions are there (policies regarding schedule breaks, workstations), it will be easy to move around without any hesitations…. at least without interrupting work outcomes.” (P3, M, 42 years, SMART-HC)* |
|  |  |  | *(Q31) “In case you have a job where you have to sit for …. say 9 hours, 10 hours, the management should be flexible in allowing for breaks or exercise or gym provisions” (P11, F, 34 years, TRADE-LC).* |
|  |  |  | *(Q32) “If you want such policy (promoting health in organisations), strong individuals should be there in that position, who can implement it, and actually see the working process get on. Unfortunately, no one is there to promote such initiatives at in larger level??….” (P11, F, 34 years, TRADE-LC).* |
|  |  |  | *(Q33) “present organisations are not flexible enough,,,,they are still rigid…. organisations may think you are lazy and neglect the work …. It may take long to implement such policies in India” (P3, M, 42 years, SMART-HC)* |
|  | Social norm/culture |  | *(Q34) “….. during meetings, it’s not a culture to even stand and I use to ignore the prompts…. I cannot stand and take notes isn’t it?. Regular desks allow me only for sitting. It (organisational policies) is rigid here…..(P16, F, 44 years, SMART-LC)* |
|  |  |  | *(Q35) “it could be the professional or personal life and we forget to take care of ourselves.* *So that is the main moto for me to go for it (intervention). So, we being Indians, we always you know take everything into ‘Just OK, let it go, let it be’ .... Yeah, taken for granted (especially our health).” (P9, F, 38 years, SMART-HC)* |
|  |  |  | *(Q36) “…. This is the work culture here………………..I knew few IT companies that allow their office workers to have recreational activities in between…. But here it is different and difficult…..” (P6, F, 39, SMART-LC)* |
|  | Environmental restructure |  | *(Q37)* *“providing exercise facilities at workplace may encourage more rather than simple break schedules… I am bored”* (*P5, M, 39 years, TRADE-LC)* |
|  |  |  | *(Q38) “Before joining the present institution, I have worked in the corporate sector…. We do get a chance to go and use the gyms or a swimming pool in the working hours…..” (P2, F, 36 years, SMART-HC)* |
|  |  |  | *(Q39) “to modify the workstation (treadmill, standing desks when probed) ……. I feel……...its waste of resources as it won’t work in our workplaces…... I bought treadmill, very expensive one for my home…..I used for only one week….. Now it (treadmill) used for stacking and cloths and utensils (laughs). At workplace……during peak of working hours, the administration of treadmill or standing desks will not work and last for long” (P7, M, 36 years, SMART-LC)* |
| Enablers to intervention | | | |
| Individual level | Opportunity | *(Q40) “……. (prompts) notifications are fine because I carry my mobiles everywhere, however, I was able to do (break sitting) only when I am free and alone” (P12, F, 36, SMART-HC)* | |
|  |  | *(Q41) “now everyone uses the smartphone… these reminders may create a habit and health benefits in long term…. (*P*7, M, 36 years, SMART group-LC)* | |
|  |  | *(Q42) “I joined the study see whether my body is healthy at present (“where I am now”) and any small movement can change my body as I hate exercise in gyms. (P6, F, 39 years, SMART-LC)”* | |
|  | Intrinsic motivation/ attitude | *(Q43) “………………..any form of exercise or simple walk or moving may be refreshing, but you know we tend to miss the activities. (P5, M, 39 years, TRADE-LC)”* | |
|  |  | *(Q44) “……………. morning, whatever happens I’ll go for at least 30 minutes’ walk at 6 AM” (P1, M, 47 years, SMART-HC)* | |
|  |  | *(Q45) “As with long sitting time during my office hours, unwanted things (backpain and mood swings) were coming back. I know some form of exercise can help me reverse them. (P2, F, 36 years, SMART-HC)”* | |
|  |  | *(Q46) “my health was not up to my expectation even with morning walk. I expected some change during the sitting time in office hours may improve my health benefits” (P1, M, 47 years, SMART-HC)”* | |
|  |  | *(Q47) “couple of times it (missed the breaks) has happened but I had made sure I need to do it.” (P2, F, 36 years, SMART-HC)* | |
|  | Intervention acceptability | *(Q48) “the smartphone break reminders…. Very interesting approach… It gives me an opportunity to move around and interact with my colleagues when reminder comes…. (Participant 11, F, 34 years, TRADE-LC)* | |
|  |  | *(Q49) “………. exercises are simple and easy than any other gym exercise I saw in social media so far…..” (P9, F, 33 years, TRADE-HC)* | |
|  |  | *(Q50) “Smartphone reminders…. Not monotonous…… different exercise not repeating on the same day…. I liked the idea and did couple of times…..I missed few times” (P1, M, 46 years, SMART-LC)* | |
|  |  | *(Q51) “Now people carry everywhere…..Its (smartphone-based reminders) fun…..one stretch based on popup…. Feels good and comfortable…..Not monotonous…… …..smartphone reminders…. different exercise not repeating on the same day…. I liked the idea and did couple of times…..I missed few times” (P1, M, 46 years, SMART-HC)* | |
|  |  | *(Q52) “you hardly get enough time during work or after work and forget to move physically……. ‘As an office staff, I sit almost entire day. We forget to take a break or stretch at least. This intervention is simple reminds me to get off the chair” (P9, F, 33 years, TRADE-HC)* | |
|  |  | *(Q53) “strongly believe physical exercise is a must….. Usually what happens is when we get involved within the work, we don't realize like, ……with the just of pop up saying you have to move, you have to stretch, that actually help at least someone is there to prompt me, this is what you have to do it right now.” (P2, F, 36 years, SMART-HC)* | |
| Interpersonal | Family support | Extrinsic motivation | *(Q54) “……My kids, even all the family members knew that I was using it and a couple of days they had reminded me getting early in the morning”” (P2, F, 36 years, SMART-HC)* |
|  |  |  | *(Q55) “…………My family was very supportive…….my family members all are kind of sports person… fitter than me” (P13, M, 42 years, SMART-HC)* |
|  |  |  | *(Q56) “……… even my husband is working in front of computer working for a large amount and even he does some kind of similar activities…” (P4, F, 43 years, TRADE-LC)* |
| Organisational | Task priority |  | *(Q57) “As Indians, we do not prioritize things (work and health balance), we take it for granted…. Only when health is at risk, they look for such interventions” (P12, F, 36, SMART-HC)* |
|  | Managerial demands |  | *(Q58) “If you want such policy (promoting physical health in organisations), strong (powerful/influential) individuals should be there in that position, who can implement it, and actually see the working process gets on. Unfortunately, no one is there to promote such in higher level…..” (P3, M, 42 years, SMART-HC)* |
|  | Policies on breaks |  | *(Q59) “……..top level organisation should be aware of how more PA will make the employee effective and productive at work. Breaking in-between sitting may actually keep us healthy and more productive” (P13, F, 37 years, SMART-LC)* |
|  | Social norm/culture |  | *(Q60)* *“these things possible only when the organisations make it compulsory for these kinds of…. practicing exercise or walk breaks…. during office hours……” (P4, F, 43, TRADE-LC)* |
|  | Environmental restructure |  | *(Q61) “After enrolling into the intervention, I have raised the level of computer so that I can stand for brief periods…… I learnt in internet”. P3, M, 42 years, SMART-HC)* |
|  |  |  | *(Q62) “similar to IT sectors, providing space and gyms may allow for long term engagement with exercise and healthy behaviours.” (P16, F, 44, SMART-LC)* |

**Supplementary file S4.** Mind map explaining the barriers and facilitators to workplace PA interventions among participants who have completed the 24 weeks of SMART-STEP trial.


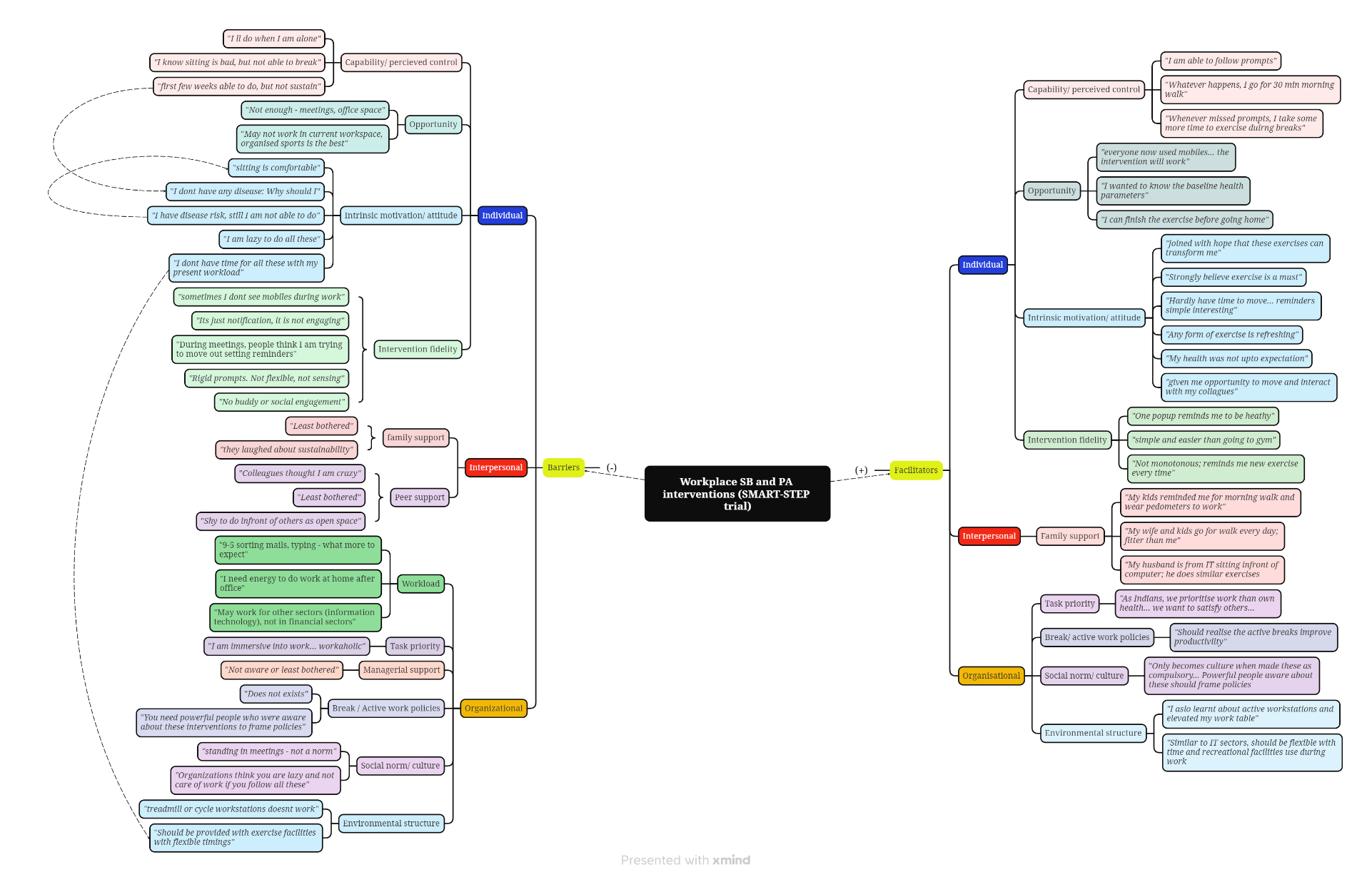

Supplement: Supplementary file 1 — Supplementary Material 1 [file 12966_2024_1661_MOESM1_ESM.docx]
